# Evaluating long non-coding RNA signatures as biomarkers for inflammatory pathway regulation and rehabilitative outcomes in Parkinson’s disease: toward a precision medicine approach

**DOI:** 10.3389/fimmu.2026.1802091

**Published:** 2026-04-20

**Authors:** Francesca La Rosa, Mario Meloni, Francesca Lea Saibene, Federica Piancone, Simone Agostini, Laura Antolini, Ivana Marventano, Ambra Hernis, Anna Salvatore, Marina Saresella, Mario Clerici

**Affiliations:** 1 IRCCS Fondazione Don Carlo Gnocchi, Milan, Italy; 2 Neurology Unit, Azienda Ospedaliero-Universitaria, Cagliari, Italy; 3 School of Medicine and Surgery, University of Milano-Bicocca, Monza, Italy; 4 Department of Pathophysiology and Transplantation, University of Milan, Milan, Italy

**Keywords:** biomarkers, cytokines, long non-coding RNA, Parkinson’s disease, rehabilitation

## Abstract

Approximately 2% of the human genome encodes proteins, while the remaining non-coding regions 19 may play critical roles in human diseases. Among these, long non-coding RNAs (lncRNAs) are 20 emerging as key regulators of gene expression through epigenetic, transcriptional, and post-21 transcriptional mechanisms, and may serve as potential biomarkers. In this pilot study, we investigated 22 lncRNAs in Parkinson’s disease (PD) patients undergoing a six-week intensive multidisciplinary 23 rehabilitation program. Plasma samples from 26 PD patients were collected before (T0) and after (T1) 24 the intervention. Array profiling of 84 inflammation- and immunity-related lncRNAs revealed that 25 86% were differentially expressed post-intervention, with 55 upregulated (fold change >2) and 17 26 downregulated. Quantitative PCR confirmed significant upregulation of MALAT1, TUG1, and XIST 27 at T1 (p < 0.05). Plasma IL-1β levels were also significantly reduced after rehabilitation (p = 0.01). A 28 2 significant reduction in UPDRS-III scores was observed at T1 (p = 0.031), reflecting improved motor 29 function; however, no significant correlation was found between clinical changes and molecular 30 findings. These preliminary, hypothesis-generating results suggest that rehabilitation may modulate 31 immune-related lncRNAs and inflammatory markers in PD, providing insights into their regulatory 32 roles in neuroinflammation and potential as biomarkers of response to rehabilitative interventions.

## 1 Introduction

Long non-coding RNAs (lncRNAs) are emerging as key regulators of gene expression in both physiological and pathological conditions, including neurodegenerative diseases such as Parkinson’s disease (PD) (1). They contribute to nuclear organization and transcriptional control by modulating transcription factors and regulating gene expression through mechanisms such as promoter activity and sense–antisense interactions. Antisense lncRNAs can regulate protein-coding genes at the same genomic locus, adding complexity to gene regulatory networks.

The human genome contains more lncRNA genes than microRNAs, underscoring their broad functional diversity. lncRNAs are involved in epigenetic and post-transcriptional regulation and can exert both activating and repressive effects. Characterizing their molecular functions is therefore critical to understanding cellular homeostasis and disease pathogenesis.

Recent studies highlight the relevance of lncRNAs in PD, showing differential expression profiles between patients and healthy controls and supporting their potential as biomarkers and modulators of disease-related pathways (2–6). Transcriptomic studies have identified lncRNAs associated with key PD-related genes, including PINK1, UCH-L1, LRRK2, and SNCA. Altered lncRNA expression has been reported in the substantia nigra and cerebellum, as well as in peripheral immune cells such as monocytes (7). lncRNAs regulate gene expression through cis- and trans-acting mechanisms, and their dysregulation may contribute to disease pathogenesis, although their precise role in PD remains to be fully defined (8–11). The detection of lncRNAs in cerebrospinal fluid (CSF)-derived exosomes further supports their potential as minimally invasive biomarkers for diagnosis and disease monitoring (3), while age-related changes in lncRNA expression may influence disease susceptibility (8, 9).

Parkinson’s disease affects approximately 2–3% of individuals over 65 and is characterized by dopaminergic neurodegeneration, α-synuclein aggregation, and chronic neuroinflammation. Beyond pharmacological treatments, structured physical activity has been shown to modulate disease progression by attenuating neuroinflammation and promoting neuronal resilience (10–12). Animal studies have further demonstrated that exercise induces neuroplastic changes, including restoration of dendritic spine density and recruitment of compensatory neural circuits (13, 14).

Despite these advances and the widespread use of clinical scales, a gap remains in identifying non-invasive, accessible peripheral biomarkers to monitor responses to rehabilitation. Here, we present an exploratory pilot study investigating the neuroimmune landscape of PD through lncRNAs. We hypothesized that inflammation-related lncRNAs may act as molecular indicators of responsiveness to rehabilitation. To this end, we profiled lncRNAs and inflammatory molecules in PD patients undergoing a six-week intensive multimodal rehabilitation program.

Although the study is limited by sample size and a single-center design, it aims to generate hypotheses and identify molecular signals that may inform future research and support the development of biomarker-driven, personalized rehabilitation strategies in PD.

## 2 Methods

### 2.1 Ethical approval

The study conformed to the ethical principles of the Declaration of Helsinki; all subjects gave informed and written consent according to a protocol approved by the local ethics committee of the Don Carlo Gnocchi Foundation (project identification code 1_16/04/2020, and subsequent amendments identified with ID 8/2021/CE_ FdG/FC/SA and with ID 101 13/2023/CE_FdG/FC/SA).

### 2.2 Patients and eligibility criteria

A total of 26 PD patients (12 males and 14 females) were consecutively recruited at the Neurology Unit of Don Carlo Gnocchi Foundation, Milan, Italy. Inclusion criteria: the diagnosis of PD was evaluated according to the Movement Disorder Society (MDS) Clinical Criteria for PD (15). Participants aged between 50 and 85 years, modified Hoehn & Yahr (mH&Y) (16) (stage from 1.5 to 3) and stable pharmacological treatment in the previous 4 weeks (17). Exclusion criteria: individuals were excluded if they had vascular, familial, or drug-induced forms of parkinsonism; any known or suspected alternative causes of parkinsonism (e.g., metabolic disorders, brain tumors); or clinical features suggestive of atypical parkinsonism. Additional exclusion criteria included the presence of significant comorbidities and/or severe systemic diseases that could contraindicate participation in an exercise program (e.g., recent surgical procedures, unstable cardiac conditions, anemia, hepatic dysfunction, pulmonary disease, chronic renal failure); sensory impairments (auditory, visual, or vestibular); a previous diagnosis of psychiatric disorders; or a clinical diagnosis of dementia, defined as a corrected Montreal Cognitive Assessment (MoCA) (18) (available at www.mocatest.org) score <15.5. Additional exclusion criteria included recent rehabilitative treatment within the past four weeks, concurrent participation in other potentially interfering clinical studies, or ongoing therapies that could conflict with the study protocol (17).

### 2.3 Clinical assessments

The following clinical scales were administered at baseline (T0) and upon completion of the rehabilitation program (T1): the Modified Hoehn and Yahr (mH&Y) scale (16) for disease staging, the Montreal Cognitive Assessment (MoCA) (18) for global cognitive function, the Movement Disorder Society-Unified Parkinson’s Disease Rating Scale Part III (MDS-UPDRS-III) (19, 20) for motor symptom severity, the Activities of Daily Living (ADL) and Instrumental Activities of Daily Living (IADL) (21) scales for functional autonomy, and the Parkinson’s Disease Questionnaire-39 (PDQ-39) (22, 23) for health-related quality of life.

### 2.4 Setting and rehabilitation program

The rehabilitation protocol, as outlined in the study protocol by Saibene et al., 2024 (18), spans a total duration of six weeks and comprises 30 outpatient sessions scheduled Monday through Friday. Each session is 160 minutes in length on three days per week (including 80 minutes of motor therapy, 40 minutes of cognitive therapy, and 40 minutes of speech therapy) and 180 minutes on two days per week (comprising 80 minutes of motor therapy, 60 minutes of cognitive therapy, and 40 minutes of speech therapy). Motor Rehabilitation: the motor component consists of 60 physical therapy sessions, each lasting 40 minutes, delivered as two sessions per day. Eighteen of these sessions incorporate “circuit training, “ combining aerobic treadmill walking and task-oriented balance exercises, administered three times per week. During these sessions, participants engage in 20 minutes of treadmill walking at moderate-to-high intensity (Rate of Perceived Exertion (RPE) scale), followed by 20 minutes of dynamic stability and balance training. The remaining 42 sessions are tailored to individual clinical needs and include resistance training, functional mobility exercises, and dual-task training. Cognitive Rehabilitation: this individualized program, supervised by a neuropsychologist, targets executive functions, attention, memory, and language. Cognitive training includes 40-minute sessions 3 times a week and 60-minute sessions with a semi-immersive Virtual Reality Rehabilitation System (VRRS) 2 times a week. Each VRRS session features six 10-minute exercises, with progress monitored through pre- and post-training assessments. Speech Therapy: Participants receive individualized treatment for voice, articulation, and swallowing issues, lasting 40 minutes per session, 5 times a week. Innovative assessment techniques and biofeedback are utilized, along with counseling for proper swallowing techniques for patients and caregivers.

### 2.5 Plasma sample collection and citokines quantification

Plasma was collected at T0 and T1 and stored at -80 °C until using. IL-1β (cod: SPCKB-PS-000216) e TNF (cod: SPCKB-PS-002803) was quantified by ELLA assay (Biotechne) according to the manufacturer’s instructions (http://www.proteinsimple.com/ella.html). Reagents for the Simple Plex™ Ella microfluidic platform (Protein Simple, CA, USA) were custom-made. Unfiltered plasma samples were centrifuged to remove any debris and diluted with the diluents provided by the manufacturer; assays were carried out following the manufacturer’s instructions. In brief, 50 μl of the diluted plasma was added to the designated cartridge, which was then inserted into the Ella instrument, requiring no additional user steps. Each cartridge contained an integrated, lot-specific standard curve, and samples were processed in triplicate internally. This setup was achieved through the presence of three nanorods in each channel corresponding to a specific biomarker, with each nanorod coated with biomarker-specific capture monoclonal antibodies. Detection antibodies and streptavidin-DyLight650 conjugate, along with washing procedures, were automatically handled by the device. Each cartridge could process sixteen samples, with 5–6 cartridges run daily, and all analyses were completed within six days. Raw data were analyzed using the SimplePlex Explorer software.

### 2.6 RNA extraction and lncRNAs expression

Total RNAs were semi-automatically extracted from plasma by QIAcube connect (Qiagen, Hilden, Germany) from 200 µL of plasma using a column-based kit (cat N° 217204) according to the manufacturer’s instructions (RNeasy Mini kit, Qiagen). Quantification of RNAs was performed using the Qubit 4 Fluorometer and Qubit RNA Assay kit (Thermo Fisher, Foster City, CA, USA). Each sample was transcribed in complementary DNA (cDNA) by RT Kit (Qiagen) in a total volume of 10 µL. Preamplification of cDNA for pathway-specific lncRNAs was applied; during the amplification step, the RT2 lncRNA PreAMP Primer Mix enables amplification of cDNA specific for the genes targeted by the RT2 lncRNA PCR Array (LAHS-004ZD, Qiagen). Preamplified cDNA is then ready for PCR array analysis using the appropriate RT2 lncRNA PCR Array (Qiagen).

### 2.7 Array-based quantitative PCR

Pre amplified cDNA samples were pulled in two separated mix and assayed for determining the expression profiles of lncRNAs by Human Inflammatory Response & Autoimmunity array (Qiagen). For PCR array analysis, 102 μl pre amplified cDNA was mixed to 1275 μl of RT^2^ SYBR^®^ Green Mastermix (Qiagen) and 1173 μl of RNase-free water according to the manufacturer’s instructions. The mixture was aliquoted into the wells of an RT2 lncRNA PCR Array, which contains pre dispensed gene-specific primer pairs. Amplification was run on an PCR CFX 9000 (Bio-Rad, Hercules, CA, US), relative gene expression is determined using the ΔΔCq method. Thresholds were determined manually for each experiment, according to the negative controls and the housekeeping genes included in the array plate. Normalization to reference genes ACTB and RPLP0 (24) as well as normalization to global mean were used. Heat maps were generated using TIGR Multi-Experiment Viewer (MeV) v4.9 (25).

The Human Inflammatory Response & Autoimmunity RT² lncRNA PCR Array profiles the expression of 84 lncRNAs that are verified or predicted to regulate proinflammatory and anti-inflammatory genes. These lncRNAs were selected based on bioinformatics predictions and experimental evidence for their potential role in modulating inflammatory responses and immune-related processes. The full list of lncRNAs included in the array is provided in **Table 1**.

**Table 1:** List of long non-coding RNA array plates.

Gene symbol	Gene name
A2ML1	A2ML1 antisense RNA 1
ABCA11P	ATP-binding cassette, sub-family A (ABC1), member 11, pseudogene
AC000120,7	AC000120.7
AC007228.9	AC007228.9
AC016629.8	Uncharacterized LOC100131691
AC068196.1	AC068196.1
AC104820.2	Uncharacterized LOC101927156
CEP83-AS1	CEP83 antisense RNA 1 (head to head)
CROCCP2	Ciliary rootlet coiled-coil, rootletin pseudogene 2
CTC-444N24. 11	CTC-444N24.11
CTC-487M23. 5	CTC-487M23.5
CTD-3185	CTD-3185P2.1
DLEU2	Deleted in lymphocytic leukemia 2 (non-protein coding)
EPB41L4A-AS1	Non-protein coding RNA 219
ERICH1-AS1	ERICH1 antisense RNA 1
FAM211A-AS1	Non-protein coding RNA 188
FGD5-AS1	FGD5 antisense RNA 1
FGF14-IT1	Hypothetical LOC283480
FLJ31306	Hypothetical LOC379025
FOXN3-AS2	PRO1768
GAS5	Growth arrest-specific 5 (non-protein coding)
GAS5-AS1	GAS5 antisense RNA 1
GRM5-AS1	GRM5 antisense RNA 1
HCG11	HLA complex group 11 (non-protein coding)
HCG18	HLA complex group 18 (non-protein coding)
HNRNPU-AS1	HNRNPU antisense RNA 1
HOTAIR	Hox transcript antisense RNA (non-protein coding)
HTR4-IT1	HTR4 intronic transcript 1 (non-protein coding)
IQCF5-AS1	IQCF5 antisense RNA 1
JPX	JPX transcript, XIST activator (non-protein coding)
LINC00094	Long intergenic non-protein coding RNA 94
LINC00116	Non-protein coding RNA 116
LINC00293	Long intergenic non-protein coding RNA 293
LINC00324	Non-protein coding RNA 324
LINC00338	Long intergenic non-protein coding RNA 338
LINC00421	HCG2019585-like
LINC00635	Hypothetical LOC151658
LINC00657	Long intergenic non-protein coding RNA 657
LINC00662	Long intergenic non-protein coding RNA 662
LINC00667	Hypothetical LOC339290
LL22NC03-N 27C7.1	LL22NC03-N27C7.1
LOC653160	Uncharacterized LOC653160
LRRC37BP1	Leucine rich repeat containing 37B pseudogene 1
MALAT1	Metastasis associated lung adenocarcinoma transcript 1 (non-protein coding
MCM3AP-AS1	MCM3AP antisense RNA 1 (non-protein coding)
MEG3	Maternally expressed 3 (non-protein coding)
NAV2-AS5	NAV2 antisense RNA 5
NCBP2-AS2	Hypothetical LOC152217
NEAT1	Nuclear paraspeckle assembly transcript 1 (non-protein coding)
NUTM2A-AS1	NUTM2A antisense RNA 1
OIP5-AS1	OIP5 antisense RNA 1
PDXDC2P	Pyridoxal-dependent decarboxylase domain containing 2, pseudogene
RMST	Rhabdomyosarcoma 2 associated transcript (non-protein coding)
RP11-1134I1 4.8	Patched 1 pseudogene
RP11-282O1 8.3	RP11-282O18.3
RP11-29G8.3	RP11-29G8.3
RP11-325K4.3	RP11-325K4.3
RP11-363E7.4	RP11-363E7.4
RP11-363G2,4	RP11-363G2,4
RP11-367N1 4.3	RP11-367N1 4.3
RP11-38P22.2	RP11-38P22.2
RP11-399K21.11	Uncharacterized LOC101929189
RP11-473I1.10	RP11-473I1.10
RP11-473M2 0.16	RP11-473M20.16
RP11-498C9. 15	RP11-498C9. 15
RP11-549J18.1	RP11-549J18.1
RP11-819C2 1.1	RP11-819C2 1.1
RP11-84C13.1	RP11-84C13.1
RP11-96D1. 10	RP11-96D1. 10
RP1-239B22. 5	RP1-239B22. 5
RP6-24A23.7	RP6-24A23.7
SDCBP2-AS1	SDCBP2 antisense RNA 1
SENP3-EIF4A1	SENP3-EIF4A1 readthrough (NMD candidate)
SIK3-IT1	SIK3 intronic transcript 1 (non-protein coding)
SLC7A11-AS1	SLC7A11 antisense RNA 1
SNHG11	Small nucleolar RNA host gene 11 (non-protein coding)
SNHG16	Small nucleolar RNA host gene 16 (non-protein coding)
SNHG5	Small nucleolar RNA host gene 5 (non-protein coding)
SNHG7	Small nucleolar RNA host gene 7 (non-protein coding)
TP73-AS1	TP73 antisense RNA 1 (non-protein coding)
TUG1	Taurine upregulated 1 (non-protein coding)
XIST	X (inactive)-specific transcript (non-protein coding)
ZFAS1	ZNFX1 antisense RNA 1
ZNRD1-AS1	ZNRD1 antisense RNA 1 (non-protein coding)
ACTB	Actin, beta

### 2.8 Droplet digital PCR for single long- non coding expression

Quantification of ABCA (cod number: 330701 LPH18089A), GAS5 (cod number: 330701 LPH11340A), MALAT1 (cod number: 330701 LPH18065A), MEG3 (cod number: 330701 LPH02974A) TUG1 (cod number: 330701 LPH18394A) and XIST (cod number:330701 LPH08103A) (Qiagen) was performed by droplet digital PCR (qPCR QX200, Bio-Rad, Hercules, CA, USA), whose assays were optimized by using serial dilutions of cDNA for each target, different primer concentrations and annealing temperatures. qPCR analysis was carried out using QuantaSoft software, version 1.7.4.0917 (Bio-Rad), and QX Manager, version 1.2 (Bio-Rad). In order to quantify copies of lncRNAs, fluorescence intensity was employed to distinguish between negative and positive droplets. Positive controls as well as negative controls were included in each experiment. Thresholds were manually determined based on the negative controls, which included a no-template control. Only samples that produced more than two positive droplets and fell above a minimum amplitude threshold were considered positive. Briefly, 3 µL of cDNA (1 ng) diluted 1:10 was mixed with lncRNA-specific primers (Qiagen) and qPCR EvaGreen Supermix (Bio-Rad). According to the manufacturer’s instructions, cDNAs were emulsified by an automatic droplet generator (QX200 AutoDG droplet generator, Bio-Rad). PCR amplification was performed using a T100 thermal cycler (Bio-Rad) as follows: 10 min at 95 °C, 40 cycles at 94 °C for 30 s and at 60 °C for 60 s, followed by 10 min at 98 °C and a hold at 4 °C. The 96-well plate was then transferred to a QX200 droplet reader (Bio-Rad). Each well was queried for fluorescence to determine the number of positive events (droplets), and the results were displayed as dot plots. The lncRNA concentration was expressed as copies/ul.

### 2.9 Statistical analysis

Changes in molecular parameters, including lncRNA expression levels (GAS5, MALAT1, XIST, TUG1, MEG3, ABCA) and plasma cytokines (IL-1β, TNF), as well as clinical outcomes (MDS-UPDRS Part III, mHY, MoCA, ADL, IADL, PDQ-39), were assessed at baseline and post-rehabilitation. Continuous variables were summarized by assessment time and visualized with spaghetti plots depicting individual patient trajectories. Differences between time points were analyzed using a mixed-effects linear model with a random intercept for each patient, with both molecular and clinical variables considered as dependent variables and time as the independent variable. Results are reported as model estimates with standard errors, z values, p values, and 95% confidence intervals.

Demographic variables were summarized descriptively. Parametric data were assessed for normality using the Shapiro–Wilk test and are reported as mean ± standard deviation.

Clinical relevance for motor function was evaluated using the Minimal Clinically Important Difference (MCID) for the MDS-UPDRS Part III, defined as a reduction of ≥3.25 points (26). The proportion of participants achieving this threshold was calculated to identify meaningful motor improvements.

Exploratory Spearman’s rank correlations were performed to investigate potential associations between changes in molecular parameters and ΔUPDRS-III. Given the small sample size, covariate-adjusted models were not performed. A significance level of 0.05 was applied for all analyses, which were conducted using STATA version 17.

## 3 Results

### 3.1 Patients disposition

A total of 26 patients (12 males, 14 females) were included in the study. The mean age was 70.3 ± 6.5 years, mean years of education 12.3 ± 4.4, and mean disease duration 7.5 ± 3.1 years. Detailed demographic characteristics are presented in **Table 2**.

**Table 2:** Demographic features of PD patients enrolled in the study.

Variable	N	Mean (years)	SD
Age	26	70,34615	6,486554
Years of education	26	12,30769	4,416055
Disease duration	26	7,554231	3,193898
Gender (M:F)	14:12		

N, Number of observations or subjects considered for this variable; Mean, the average of the variable’s values expressed in years; SD, standard deviation.

**Figure 1:**
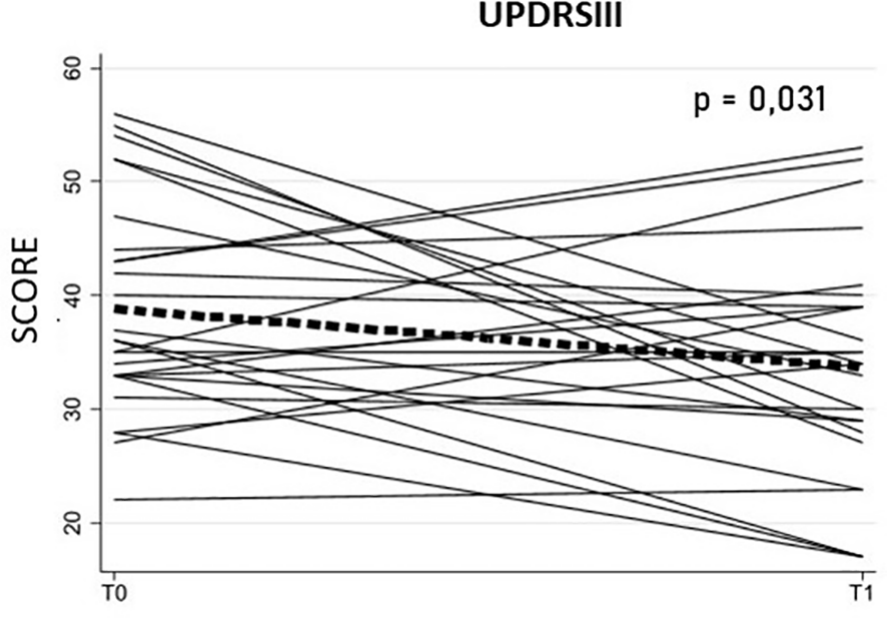
UPDRS-III (Unified Parkinson’s Disease Rating Scale – Part III) scores at baseline (T0) and after rehabilitation (T1) in Parkinson’s disease (PD) patients (N = 26). Each line represents an individual participant’s motor trajectory over time (spaghetti plot). The bold red line shows the average score at each time point, and the shaded area represents the range across participants. Higher scores indicate more severe motor symptoms. Panel A highlights the statistically significant reduction in UPDRS-III from T0 to T1 (p = 0.031).

Following six weeks of structured rehabilitation, significant improvement was observed in UPDRS-III scores, which decreased by 5.12 points (Estimate = -5.12, SE = 2.39, p = 0.032, 95% CI: -9.79 to -0.44), indicating better motor performance in PD patients, as lower scores reflect improved function (**Figure 1**). Other clinical measures showed non-significant trends toward improvement: ADL (Estimate = -0.35, p = 0.10) and IALD (Estimate = -0.54, p = 0.077) decreased slightly, while MHY (Estimate = -0.10, p = 0.34), MOCA (Estimate = -0.12, p = 0.91), and QOL (Estimate = -0.80, p = 0.63) remained essentially unchanged. Clinical outcomes are summarized in **Table 3**.

**Table 3:** Estimated (regression coefficient ) for time effect (T1 vs T0) in Clinical Data of PD Patients following rehabilitation.

Variable	N	Estimate	Std. error	z	p	95% CI lower	95% CI upper
UPDRS-III	26	-5.1154	2.3854	-2.1444	**0.0320**	-9.7908	-0.4400
mH&Y	26	-0.1000	0.1045	-0.9571	0.3385	-0.3048	0.1048
MoCA	26	-0.1154	1.0481	-0.1101	0.9123	-2.1697	1.9389
ADL	26	-0.3462	0.2103	-1.6463	0.0997	-0.7583	0.0659
IALD	26	-0.5385	0.3040	-1.7714	0.0765	-1.1342	0.0573
PDQ-39	26	-0.8002	1.6730	-0.4783	0.6324	-4.0792	2.4788

Std. Error, standard error of the estimate; z, z-statistic; p, p-value; 95% CI, 95% confidence interval.

PD, Parkinson’s Disease;

UPDRS-III, Unified Parkinson’s Disease Rating Scale Part III;

mH&Y, Hoehn and Yahr,

MoCA, Montreal Cognitive Assessment,

IADL, Instrumental Activities of Daily Living,

ADL, Activities of Daily Living,

PDQ-39, Parkinson’s Disease Questionnaire-39.

### 3.2 Plasma lncRNA expression and inflammation dynamics in PD patients

**Figure 2:**
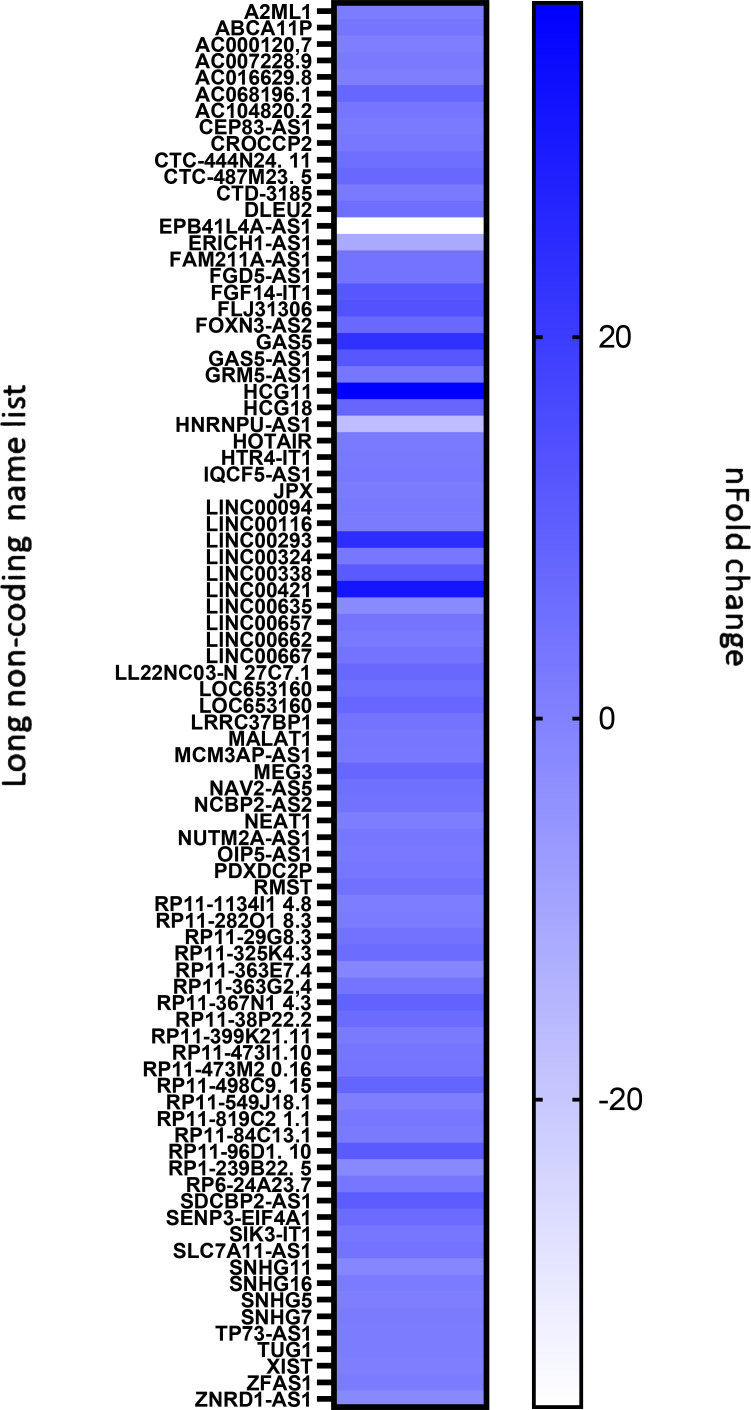
Expression profiles of 84 inflammation- and immunity-related long non-coding RNAs (lncRNAs) in plasma from PD patients (N = 26), assessed using the Human Inflammatory Response & Autoimmunity RT² PCR Array. The heatmap displays log2 fold changes in lncRNA expression between baseline (T0) and post-rehabilitation (T1), with a color gradient ranging from -20 (light blue, downregulation) to +20 (dark blue, upregulation). All abbreviations used for lncRNAs are defined in **Table 1**.

As an exploratory analysis, the plasma expression of 84 long non-coding RNAs (lncRNAs) was assessed in PD patients at baseline (T0) and after six weeks of structured rehabilitation (T1) using a qPCR array, normalized to the global mean of housekeeping genes. Among these, 55 lncRNAs showed a fold change (FC) > 2 at T1 (upregulated), whereas 17 lncRNAs were downregulated (FC < 2) (**Figure 2**). Six lncRNAs—ABCA, GAS5, MALAT1, MEG3, TUG1, and XIST—were selected for further analysis based on prior evidence linking them to inflammation, Parkinson’s disease, or other neurodegenerative conditions.

**Figure 3:**
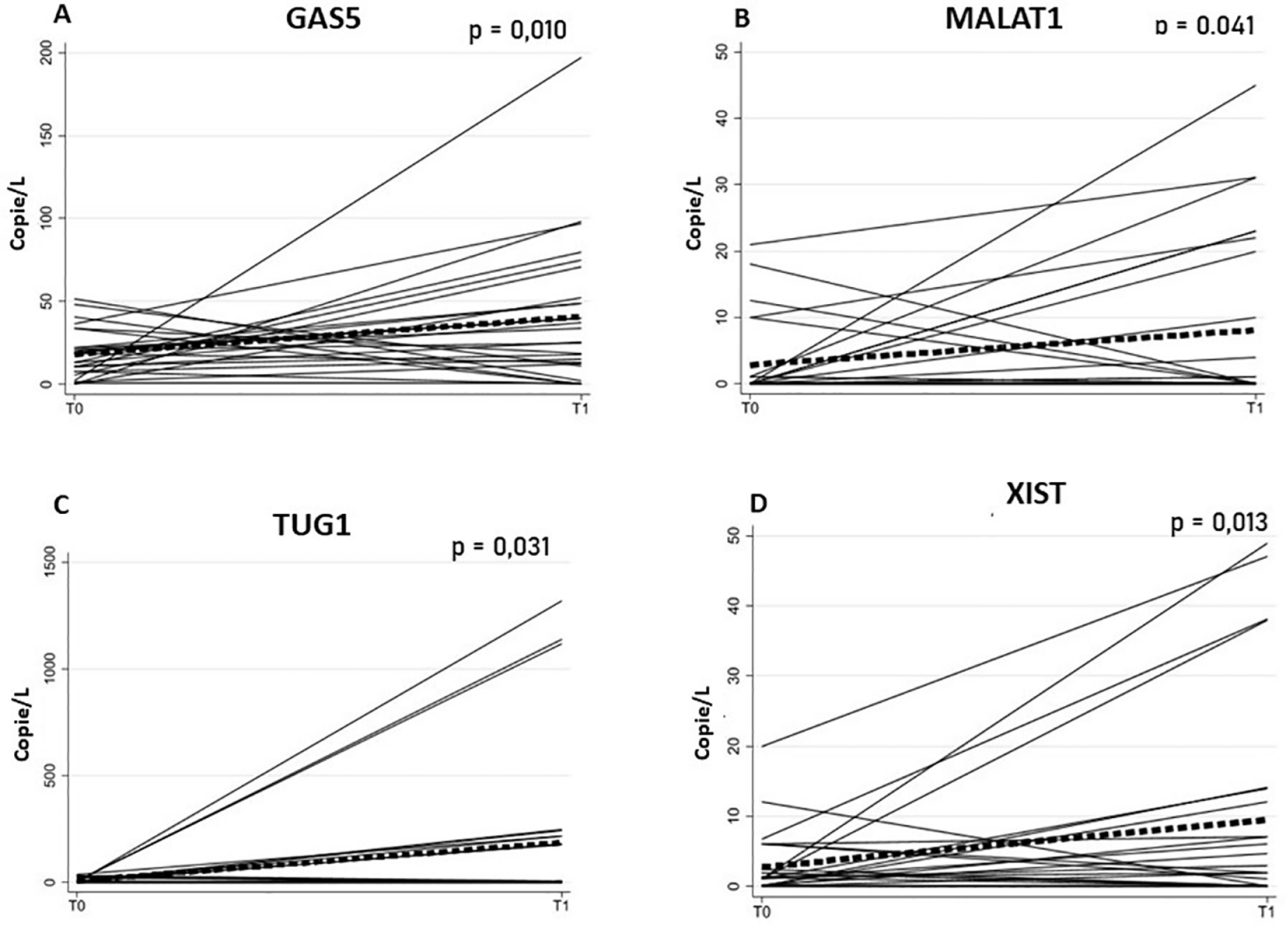
Plasma concentrations of selected long non-coding RNAs (lncRNAs) GAS5 **(A)**, MALAT1 **(B)**, TUG1 **(C)**, and XIST **(D)** in PD patients (N = 26) at baseline (T0) and after rehabilitation (T1). Each line represents an individual trajectory over time (spaghetti plot). The bold red line shows the average trajectory, and the shaded area indicates the range across participants. Concentrations are expressed as copies/ng (nanograms RNA). P-values indicate the significance of changes between T0 and T1 for each lncRNA.

To further quantify these changes, mixed-effects regression analyses were performed for each selected lncRNA, providing estimates of the effect of time (T1 vs T0) with standard errors, z-statistics, p-values, and 95% confidence intervals. Significant increases were observed for XIST (6.75, SE = 2.72, z = 2.48, p = 0.013, 95% CI: 1.41–12.09), GAS5 (22.57, SE = 8.85, z = 2.55, p = 0.011, 95% CI: 5.21–39.92), MALAT1 (5.25, SE = 2.61, z = 2.01, p = 0.044, 95% CI: 0.13–10.37), and TUG1 (179.50, SE = 73.45, z = 2.44, p = 0.015, 95% CI: 35.54–323.46) (**Figure 3**), whereas ABCA (1.54, SE = 1.06, z = 1.45, p = 0.146, 95% CI: -0.54–3.62) and MEG3 (3.54, SE = 2.50, z = 1.42, p = 0.156, 95% CI: -1.35–8.44) showed non-significant trends. All model estimates and associated statistics are reported in **Table 4**.

**Table 4:** Estimated changes in long non-coding RNA expression from baseline (T0) to post-rehabilitation (T1) in PD patients.

lncRNA	Estimate	Std. error	z value	p value	95% CI min	95% CI max
XIST	6.7481	2.7247	2.4767	0.0133	1.4078	12.0883
GAS5	22.5692	8.8549	2.5488	0.0108	5.2140	39.9245
ABCA	1.5404	1.0594	1.4540	0.1459	-0.5360	3.6168
MALAT	5.2492	2.6118	2.0098	0.0445	0.1302	10.3683
TUG	179.500	73.4517	2.4438	0.0145	35.5373	323.4627
MEG3	3.5442	2.4981	1.4187	0.1560	-1.3520	8.4405

Std. Error: standard error of the estimate z: z-statistic p: p-value 95% CI: 95% confidence interval

PD, Parkinson’s Disease;

XIST, (X-inactive specific transcript),

GAS5, (growth arrest-specific 5),

MALAT1, (metastasis-associated lung adenocarcinoma transcript 1).

TUG1, (Taurine Upregulated Gene 1).

ABCA, (ATP-binding cassette subfamily A member).

MEG3, (maternally expressed gene 3).

**Figure 4:**
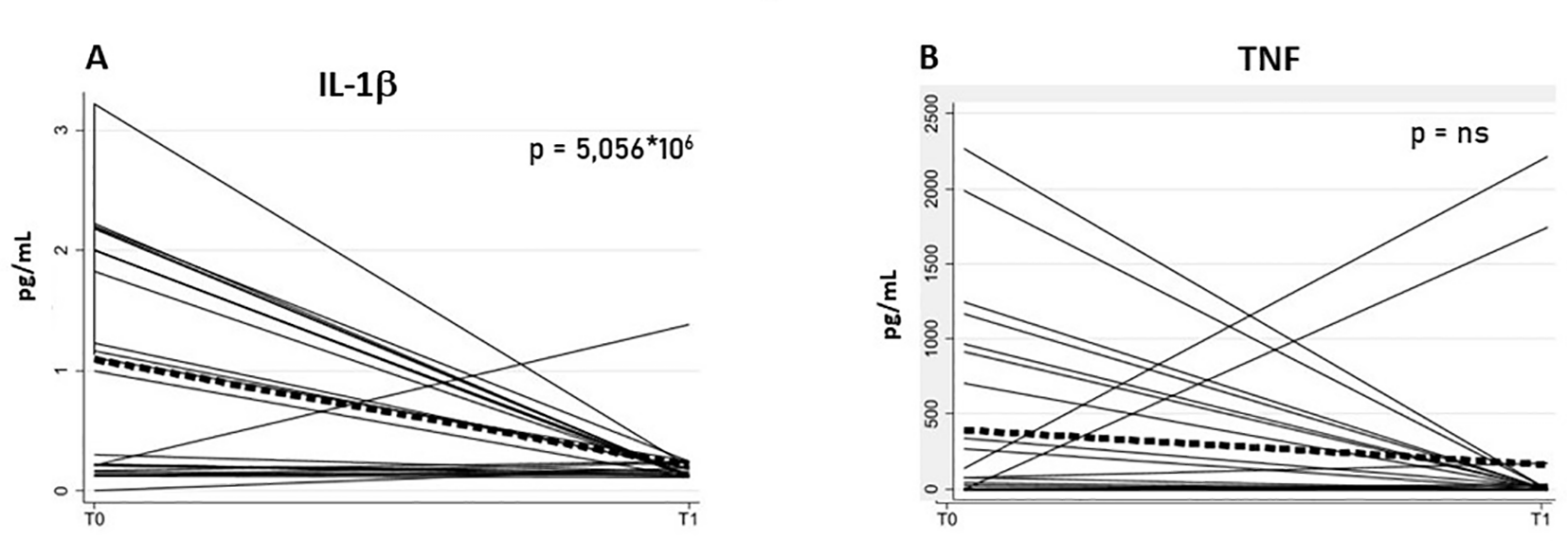
Plasma concentrations of the inflammatory cytokines IL-1β **(A)** and TNF **(B)** in PD patients (N = 26) at baseline (T0) and after rehabilitation (T1). Each line represents an individual trajectory over time (spaghetti plot). The bold red line shows the average trajectory, with the shaded area representing the range across participants. Cytokine concentrations are expressed in pg/mL (picograms per milliliter). Panel A (IL-1β) shows a statistically significant reduction from T0 to T1 (p = 5.056 × 10⁻^6^).

Similarly, analysis of inflammatory markers revealed a significant reduction in IL-1β (Interleukin-1 beta) by -0.88 (SE = 0.19, z = -4.56, p < 0.0001, 95% CI: -1.26–0.50), whereas TNF (Tumor Necrosis Factor) decreased by -230.85 (SE = 162.28, z = -1.42, p = 0.155, 95% CI: -548.91–87.20), a non-significant change (**Figure 4**). Model estimates and associated statistics are reported in **Table 5**.

**Table 5:** Regression estimates of plasma inflammatory markers in PD patients following rehabilitation.

Marker	Estimate	Std. E	z	p	95% CI lower	95% CI upper
IL-1β	-0.8824	0.1934	-4.5624	5.06e-06	-1.2615	-0.5033
TNF	-230.8523	162.2762	-1.4226	0.1549	-548.907	87.2033

Std. Error, standard error of the estimate; z, z-statistic; p, p-value; 95% CI, 95% confidence interval.

PD, Parkinson’s Disease;

IL-1β, Interleukin-1 beta.

TNF, Tumor Necrosis Factor.

These results provide preliminary evidence that structured rehabilitation may influence both up- and downregulated immune-related lncRNAs and inflammatory mediators in PD patients. Given the exploratory nature of this study, these findings serve as a basis for future research aimed at evaluating the potential of these molecules as biomarkers of immune adaptation and for informing rehabilitative strategies.

### 3.3 Correlation between molecular and clinical parameters

To explore potential associations between clinical improvement and molecular changes, Spearman’s correlation analyses were conducted between the change in MDS-UPDRS-III scores (ΔUPDRS-III, T1–T0) and the expression changes of selected lncRNAs (ΔGAS5, ΔMALAT1, ΔXIST, ΔTUG1, ΔMEG3, ΔABCA) as well as plasma cytokine levels (ΔIL-1β, ΔTNF).

No statistically significant correlations were found between ΔUPDRS-III and any of the molecular markers analyzed (p > 0.05 for all comparisons). Similarly, changes in lncRNA expression did not correlate significantly with IL-1β or TNF levels.

## 4 Discussion

Long non-coding RNAs (lncRNAs) play important roles in immune regulation by influencing the development, differentiation, and activation of myeloid (macrophages, monocytes, dendritic cells) and lymphoid (NK cells, T and B lymphocytes) immune cells (27). Their expression is linked to immune cell growth and specialization, and they may modulate immune responses during inflammation by acting as microRNA sponges or precursors, affecting mRNA stability and signaling pathways (28, 29). Dysregulation of lncRNAs has been associated with various diseases, including neurodegenerative disorders. Several studies have shown that lncRNA expression differs between PD patients and healthy controls, highlighting their potential as biomarkers and therapeutic targets (2–6). However, their comprehensive functional roles remain to be fully elucidated.

In the context of neurodegeneration, lncRNAs are highly expressed in the central nervous system and may contribute to neuroinflammation and oxidative stress, features relevant to Parkinson’s disease (PD) (3, 30, 31). Changes in lncRNA levels have also been suggested to relate to disease severity (32), and in cellular models, alpha-synuclein, a key PD protein, can modify the expression of several lncRNAs (33). Lifestyle factors, including exercise, have been shown to influence lncRNA expression, and in a mouse model of PD, aerobic exercise was associated with improved behavior and specific changes in lncRNA levels (34).

The present pilot study explored modulation of lncRNAs in PD patients participating in a structured outpatient rehabilitation program within the Complex Outpatient Macroactivity (MAC) framework. Neuroinflammation is a recognized feature of PD, with increased concentrations of inflammatory proteins, including cytokines and chemokines, in cerebrospinal fluid and plasma (35–37). IL-1β and TNF are two key inflammatory cytokines elevated in PD (37).

Previous studies suggest that rehabilitation and lifestyle interventions can modulate inflammatory pathways and related molecular mediators, although data in PD are limited. In our exploratory study, both IL-1β and TNF were reduced following rehabilitation, with IL-1β showing a statistically significant decrease. Six lncRNAs previously associated with PD pathogenesis—GAS5, MALAT1, XIST, TUG1, MEG3, and ABCA—were upregulated after rehabilitation, with qPCR confirming significant increases for GAS5, MALAT1, XIST, and TUG1. Changes in MEG3 and ABCA were not statistically significant.

Although UPDRS-III scores decreased from T0 to T1, indicating clinical improvement, no significant correlations were observed between molecular changes and ΔUPDRS-III. This suggests that molecular and clinical improvements occurred in parallel, without a direct association at the individual level. Previous studies have implicated GAS5, MALAT1, TUG1, and XIST in regulation of inflammation and cellular stress responses, although primarily in contexts other than Parkinson’s disease (38–50). GAS5 can act as a negative regulator of proinflammatory cytokines (38). MALAT1 modulates mitochondrial function, apoptosis, and oxidative stress (39–44); TUG1 (45, 46) and XIST (47–50) function as miRNA sponges influencing NF-κB signaling. Based on these observations, and considering our findings of upregulation of GAS5, MALAT1, TUG1, and XIST following rehabilitation, these lncRNAs may participate in immune adaptation and modulation of inflammatory pathways, although further studies are needed to confirm their functional role.

This study is among the first to report coordinated modulation of these four lncRNAs after a non-pharmacological intervention. These exploratory results provide a rationale for future studies to evaluate lncRNAs as biomarkers of immune adaptation and as potential targets for adjunctive therapies in neurodegenerative disorders.

### 4.1 Strengths and limitations

This pilot study explored a novel and under-investigated area: long non-coding RNAs, whose functional roles require further clarification. We observed a potential link between rehabilitation-induced clinical improvements and changes in lncRNA expression, although causality cannot be established. The modulation of lncRNAs may reflect, rather than directly mediate, physiological responses to rehabilitation, and the absence of a control cohort limits the ability to distinguish intervention-specific effects.

Rehabilitation was associated with modest clinical improvements in UPDRS-III scores, alongside changes in specific lncRNAs and inflammatory markers. The small sample size and individual variability likely contributed to the lack of statistically significant correlations between molecular and clinical outcomes. All participants were on stable pharmacological regimens prior to enrollment, but residual confounding from medication, disease duration, or baseline inflammatory status cannot be excluded, and covariate-adjusted analyses were not feasible. Nonetheless, parallel trends—such as upregulation of inflammation-related lncRNAs and decreased IL-1β levels in patients showing clinical improvement—support the hypothesis of a systemic response involving both molecular and functional pathways. Larger, controlled studies, ideally combined with *in vivo* experiments, are needed to determine whether these lncRNAs could serve as biomarkers or mediators of immune adaptation and neuroplasticity in PD.

## 5 Conclusions

These pilot results indicate that lncRNAs may contribute to the systemic response to rehabilitation in PD. Their role in clinical improvement remains speculative, but they hold promise as biomarkers and targets for interventions modulating neuroinflammation. Larger, mechanistic studies are needed to validate these findings and assess their integration into personalized rehabilitation strategies.

## Data Availability

The raw data supporting the conclusions of this article will be made available by the authors, without undue reservation.
